# The impact of resident training on robotic operative times: is there a July Effect?

**DOI:** 10.1007/s11701-024-01929-3

**Published:** 2024-05-10

**Authors:** Falisha F. Kanji, Eunice Choi, Kai B. Dallas, Raymund Avenido, Juzar Jamnagerwalla, Stephanie Pannell, Karyn Eilber, Ken Catchpole, Tara N. Cohen, Jennifer T. Anger

**Affiliations:** 1 Department of Surgery, Cedars-Sinai Medical Center, Los Angeles, CA USA; 2 Division of Urology, Department of Surgery, Cedars-Sinai Medical Center, Los Angeles, CA USA; 3 Division of Urology and Urologic Oncology, Department of Surgery, City of Hope, Lancaster, CA USA; 4 Division of Urology, Baylor Scott and White, Round Rock, TX USA; 5 Division of Urology, Santa Monica Urology, Santa Monica, CA USA; 6 Department of Anesthesia and Perioperative Medicine, Medical University of South Carolina, Charleston, SC USA; 7 Department of Urology, University of California, San Diego, 9400 Campus Point Drive, #7897, La Jolla, CA 92037 USA

**Keywords:** Robotic-assisted surgery, Urologic surgical procedures, Operative times, Resident training

## Abstract

It is unknown whether the July Effect (a theory that medical errors and organizational inefficiencies increase during the influx of new surgical residents) exists in urologic robotic-assisted surgery. The aim of this study was to investigate the impact of urology resident training on robotic operative times at the beginning of the academic year. A retrospective chart review was conducted for urologic robotic surgeries performed at a single institution between 2008 and 2019. Univariate and multivariate mix model analyses were performed to determine the association between operative time and patient age, estimated blood loss, case complexity, robotic surgical system (Si or Xi), and time of the academic year. Differences in surgery time and non-surgery time were assessed with/without resident presence. Operative time intervals were included in the analysis. Resident presence correlated with increased surgery time (38.6 min *(p* < *0.001)*) and decreased non-surgery time (4.6 min *(p* < *0.001)*). Surgery time involving residents decreased by 8.7 min after 4 months into the academic year (July–October), and by an additional 5.1 min after the next 4 months *(p* = *0.027,* < *0.001)*. When compared across case types stratified by complexity, surgery time for cases with residents significantly varied. Cases without residents did not demonstrate such variability. Resident presence was associated with prolonged surgery time, with the largest effect occurring in the first 4 months and shortening later in the year. However, resident presence was associated with significantly reduced non-surgery time. These results help to understand how new trainees impact operating room times.

## Introduction

Determining the organizational and systems-related factors that contribute to trends in surgical outcomes is complex and often difficult. Seasonal trends have been linked to variations in outcomes, with increased rates of post-operative infections in the summer months [1], and hospital performance being affected by weekends and holidays due to a shortage of medical staff [2–4]. Several studies hypothesize worsened outcomes for patients receiving care at teaching hospitals in July, as new trainees enter residency training and engage in patient care responsibilities for the first time. Despite better outcomes for patients treated at teaching hospitals overall compared to those at non-teaching hospitals [5], there are reported trends of increased medical errors, hospital inefficiency, and higher morbidity to be associated with the period of resident turnover—a phenomenon popularly referred to as the July Effect [6]. During this period, new residents and trainees have been known to disrupt the multidisciplinary flow of patient care, which has been shown to rely on staff experience and a high level of physician-nurse collaboration [7]. The July Effect has been observed in numerous specialties, including neurosurgery [8], pancreatic surgery [9], and urology [10]. Data from plastic surgery and several others [8, 11–14], in contrast, demonstrate a lack of significant findings to support the presumed phenomenon in July.

At teaching hospitals, urology constitutes a large proportion of robotic-assisted surgery (RAS) cases. Moreover, RAS continues to replace conventional laparoscopy in a wide range of procedures; as such, residents are increasingly participating in numerous RAS procedures. To meet the high-volume demand of RAS, residency programs have formally begun to integrate robotic surgery training into their curriculum. From 2020 to 2021 alone, the Accreditation Council for Graduate Medical Education (ACGME) increased the minimum number of robotic cases urology residents are required to perform from 50 to 80 [15]. This increase signifies the growing importance of achieving both optimal learning curves and operating room efficiency, without compromising patient safety. The heightened involvement of urology residents in RAS provides an ideal population within which to help clarify the role of a July phenomenon in robotic surgery [16, 17]. Though RAS rapidly emerges at the forefront of minimally invasive surgery, associations between resident involvement and the July Effect in RAS have not been thoroughly investigated. Therefore, the objective of this study was to investigate the July Effect in urologic RAS at a single, high-volume academic medical center.

## Materials and methods

After obtaining institutional review board (Pro00029501) approval, a retrospective chart review was conducted for robotic urological surgery cases performed at a single metropolitan tertiary medical center from March 2008 to September 2019. Patient and surgical characteristics that were collected included patient age, body mass index (BMI), time of academic year (July–October, November–February, March–June), procedure performed, any concomitant procedures, estimated blood loss (EBL), and use of da Vinci robotic console Si or Xi (Intuitive Surgical, Sunnyvale, CA). Specific operative timepoints were collected and assessed for differences: patient entrance to operating room (OR), procedure start, initial incision, incision closure, procedure stop, and patient exit from OR. Surgery time (ST) was defined as the operative duration between the initial incision and procedure stop. Non-surgery time (NT), or non-skin-to-skin OR time, was defined as the total cumulative duration from when the patient entered the OR to the initial incision, and from the procedure stop to when the patient exited the OR.

We had the unique opportunity to address the concept of the July Effect as a natural experiment due to the implementation of a urology residency at our academic center in 2011. Resident involvement in urologic cases began when our institution’s residents began working with the academic staff after the end of their internship, in July 2012. Two cohorts were constructed: cases with residents and cases without residents. After July 2012, a large proportion of cases remained uncovered by residents until a full complement of residents was present. To control for the fact that residents were introduced after an individual surgeon had amassed a certain clinical volume, we incorporated attending-specific case experience into the model.

Cases were redistributed into one of five categories to account for differences in case complexity. Category 1 (robotic-assisted laparoscopic prostatectomy (RALP)) included radical and simple prostatectomy. Category 2 (robotic-assisted sacrocolpopexy) contained only sacrocolpopexy. Category 3 (robotic-assisted cystectomy) contained only cystectomy. Category 4 (robotic-assisted nephrectomy) included radical nephrectomy, partial nephrectomy, adrenalectomy, partial retroperitoneal nephrectomy, and nephroureterectomy. Category 5 (robotic-assisted pyeloplasty) included bladder diverticulectomy, pyeloplasty, partial cystectomy, ureteral reimplantation, and ureterectomy.

### Statistical analysis

A mixed effects model was developed to estimate the independent impact of each variable on ST, with each variable as a fixed effect and the particular attending surgeon as a random effect. The multivariable models used in this study factored in age, BMI, EBL, case type, da Vinci model, and whether the case involved multiple procedures. To account for significant covariates, we included a month of the year as the interaction term in the adjusted model.

## Results

A total of 1728 urologic robotic cases were performed by 13 different attending surgeons during the study period. A total of 686 cases (39.7%) were performed independently by attendings, and 1042 cases (60.3%) were performed in the presence of surgical residents. Of these recorded cases, there were 1,361 (78.8%) RALPs, 124 (7.2%) sacrocolpopexies, 33 (1.9%) cystectomies, 150 (8.7%) nephrectomies, and 60 (3.5%) pyeloplasties (Table 1). Tables 2 and 3 present the differences in mean operative times by case category with and without resident presence. Average ST did not significantly differ by resident involvement at the univariate level (248.77 min without residents, 244.97 min with residents; *p* = *0.25)* (Table 2). NT, on the other hand, significantly improved with resident presence by 4.6 min (*p* < 0.001) (Table 3). The unadjusted relative impact of each variable on surgery time with and without residents are presented in Table 4. Factors associated with a prolonged effect on operative time in cases with residents included increasing patient age and BMI. For each increase in BMI value, ST was estimated to increase by 1.98 min (*p* < 0.001) in cases with residents. The presence of a resident was associated with a slightly reduced EBL *(p* < *0.001)*. Cystectomies (Group 3) and nephrectomies (Group 4) appeared to be significantly prolonged when residents were involved.

**Table 1:** Recoded case categories, original cases, and observed frequencies

Recoded categories	Original cases	No. (%)
Group 1—robotic-assisted laparoscopic prostatectomy (RALP)	• Prostatectomy robotic	1361 (79)
	• Simple prostatectomy	
Group 2—sacrocolpopexy	• Colpopexy	124 (7)
Group 3—cystectomy	• Cystectomy ileal conduit robotic	33 (2)
Group 4—nephrectomy	• Adrenalectomy	150 (9)
	• Robotic nephrectomy	
	• Nephrectomy partial	
	• Nephrectomy partial retroperitoneal	
	• Nephroureterectomy	
Group 5—pyeloplasty	• Bladder diverticulectomy	60 (3)
	• Robotic cystectomy partial	
	• Pyeloplasty	
	• Ureteral reimplantation	
	• Ureterectomy

**Table 2:** Mean operative times (in minutes) with and without resident for ST

Case type	With resident	Without resident	*p* value
Overall	244.97	248.77	0.250
Group 1—RALP	248.45	249.24	0.800
Group 2—sacrocolpopexy	214.53	228.33	0.480
Group 3—cystectomy	488.40	352.67	0.011
Group 4—nephrectomy	220.34	301.50	0.411
Group 5—pyeloplasty	200.10	152.71	0.002

**Table 3:** Mean operative times (in minutes) with and without resident for NT

Case type	With resident	Without resident	*p* value
Overall	58.80	61.48	< 0.001
Group 1—RALP	56.10	61.39	< 0.001
Group 2—sacrocolpopexy	55.42	62.00	0.481
Group 3—cystectomy	73.40	75.67	0.852
Group 4—nephrectomy	68.64	69.00	0.977
Group 5—pyeloplasty	66.06	61.57	0.367

**Table 4:** Multivariate analysis of unadjusted variables and their associated impact on ST for cases without resident presence and with resident presence

	Without resident presence		With resident presence	
	Effect (95% CI)	*p* value	Effect (95% CI)	*p* value
Age	− 0.43 (− 0.74, − 0.11)	0.008	0.19 (− 0.14, 0.51)	0.263
BMI	0.40 (0.06, 0.09)	0.015	1.98 (1.29, 2.68)	< 0.001
EBL	0.08 (0.06, 0.09)	< 0.001	0.05 (0.04, 0.06)	< 0.001
Case				
Group 1—RALP	Ref	Ref	Ref	Ref
Group 2—sacrocolpopexy	9.07 (− 31.88, 49.97)	0.668	− 34.79 (− 51.96, − 17.71)	< 0.001
Group 3—cystectomy	64.20 (1.00, 127.51)	0.049	225.19 (206.06, 244.27)	< 0.001
Group 4—nephrectomy	11.80 (− 63.51, 87.13)	0.76	− 26.21 (− 35.98, − 16.49)	< 0.001
Group 5—Pyeloplasty	− 76.44 (− 52.70, − 22.60)	< 0.001	− 37.63 (− 52.70, − 22.60)	< 0.001
Machine				
Si	Ref	Ref	Ref	Ref
Xi	− 23.43 (− 116.79, 36.11)	< 0.001	− 29.11 (− 31.21, − 15.33)	0.446
Time of academic year				
July–October	Ref	Ref	Ref	Ref
November–February	− 3.10 (− 11.09, 4.84)	0.448	− 8.73 (− 16.40, − 1.06)	0.027
March–June	− 1.53 (− 9.32, 6.35)	0.702	− 13.79 (− 21.23, − 6.36)	< 0.001
Multiple procedures	17.93 (5.66, 30.14)	0.004	24.82 (15.60, 33.91)	< 0.001

**Fig. 1:**
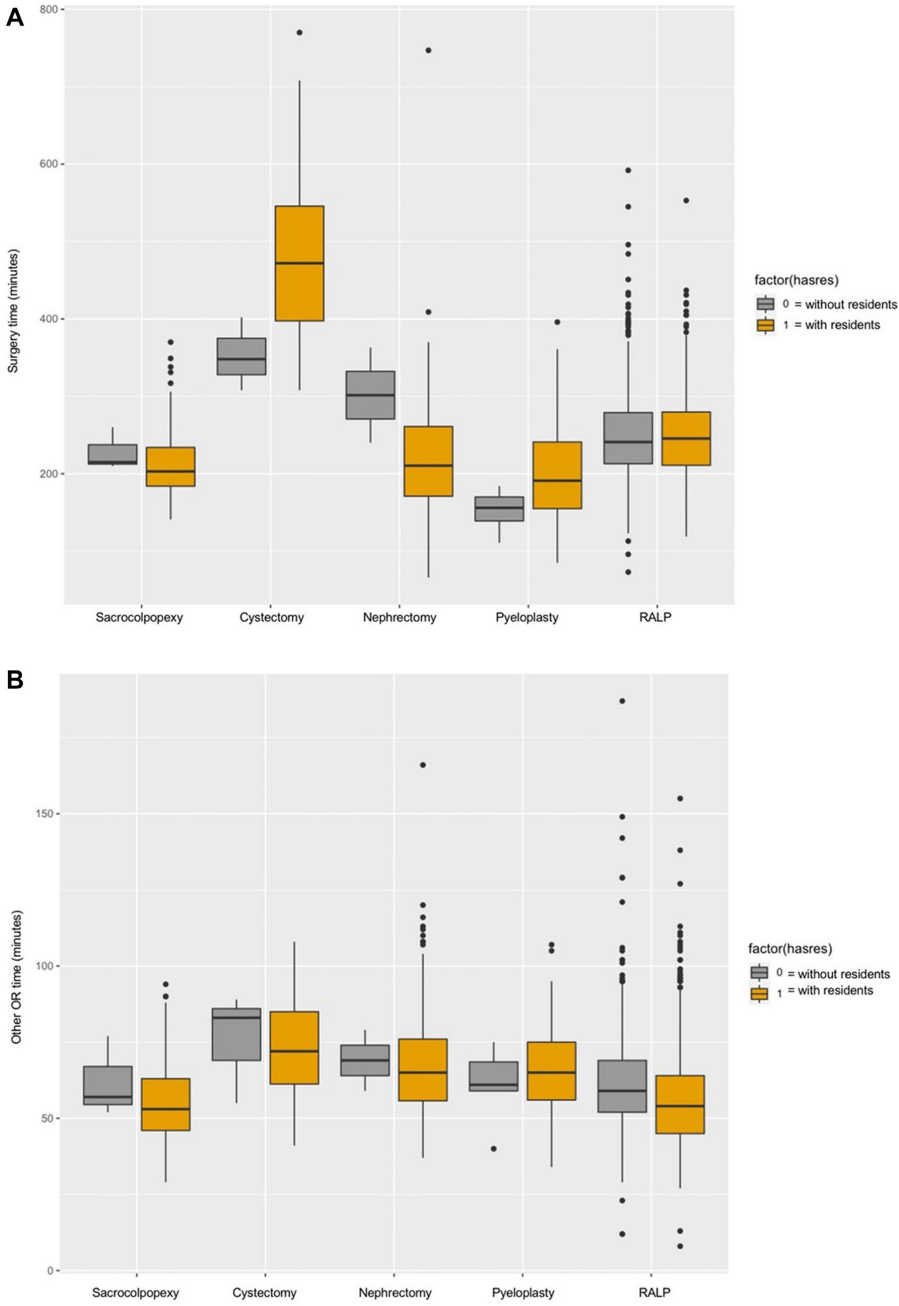
**a** Comparison of ST with and without resident presence by case type (created using the R Project for Statistical Computing). **b** NT with and without resident presence by case type (created using the R Project for Statistical Computing)

Following adjustment (Table 5), resident presence was determined to be a significant independent predictor of longer ST. Overall, resident presence was associated with increased ST by 38.62 min *(p* < *0.001)*. ST in cases involving residents was shown to decrease by 8.7 min after the first 4 months of the academic year (July–October), then by an additional 5.1 min after the next 4 months (November–February) *(p* = *0.027,* < *0.001).* When the interaction term was included in the analysis, the ST of cases with residents improved by 18.94 min on average after the first 4 months of the academic year (July–October vs November–June, *p* = *0.001*). There was significant variability to ST in cases with residents when we compared time intervals stratified by case type (Fig. 1a). Cases with attending surgeons only, in contrast, did not exhibit significant variability across case type. NT did not significantly vary across both cohorts (Fig. 1b).

**Table 5:** Multivariate analysis of the variables with adjusted impact on ST

Variable	Effect (95% CI)	*p* value
Age	0.01 (− 0.27, 0.25)	0.93
BMI	0.63 (0.35, 0.91)	< 0.001
EBL	0.07 (0.06, 0.08)	< 0.001
Case		
Group 1—RALP	Ref	Ref
Group 2—sacrocolpopexy	− 27.89 (− 43.67, − 12.28)	< 0.001
Group 3—cystectomy	211.80 (194.20, 229.29)	< 0.001
Group 4—nephrectomy	− 27.04 (-36.16, -17.95)	< 0.001
Group 5—pyeloplasty	− 44.56 (− 57.75, 31.40)	< 0.001
Machine		
Si	Ref	Ref
Xi	− 37.19 (− 48.36, − 26.06)	< 0.001
Time of academic year		
July to October	Ref	Ref
November–February	8.16 (− 0.75, 17.07)	0.074
March–June	5.78 (− 2.91, 14.48)	0.194
Multiple procedures	21.60 (14.14, 29.00)	< 0.001
Resident presence	38.62 (27.33, 49.96)	< 0.001
Interaction of time/res^a^		
July–October	Ref	Ref
November–February	− 17.43	0.003
March–June	− 18.94	0.001

^a^Interaction of time of the academic year by the presence of residents

## Discussion

This study investigated the impact of resident presence on robotic operative times in urology at a single high-volume academic hospital. We found a July Effect on operative times for urologic robotic surgery (U-RAS), as ST with residents were significantly longer between July to October. This finding expands on results from other studies that investigated the effects of resident involvement on operative efficiency throughout different periods of the academic year. In cardiac surgery, operative times averaged 295 ± 90 min *(p* < *0.05)* in surgeries performed from July to August. The rest of the academic year (September to June) demonstrated an improvement with a reduced operative time of 288 ± 90 min (*p* < *0.05)* [18]. These findings are consistent with other studies investigating the impact of resident training in urology demonstrating increased operative times and no difference in overall complication rates [10, 19–21]. The impact of resident training on minimally invasive surgery outcomes, however, remains largely unexplored.

The natural dichotomization of cases without (prior to July 2012) and with resident trainees (July 2012 and after) allowed us to explore the impact of resident presence on operative times for urologic cases. Most previous studies that investigated the July Effect did not have this advantage, thus largely assuming a causative relationship between trainee involvement and prolonged operative times [8, 18]. The study by Afshar et al. [10] is notable as their analyses of resident involvement on mid-urethral sling procedures at our institution captured the resident-specific effect due to the new implementation of the residency training program in 2011 [10]. Our data with U-RAS demonstrated consistent findings, thereby adding support to the existence of a July Effect for operative time in urologic surgery.

Despite our findings, literature from other surgical specialties (plastic surgery and lower-extremity orthopedic trauma) [11, 22] did not identify any significant seasonal differences in operative time. Our findings may reflect a more difficult learning curve for laparoscopically naïve residents and interns, which could translate to longer operative times during their initial learning curve. A study in 2003 investigating subjective incident reports across three teaching hospitals found that the most commonly cited factor for surgical errors was inexperience/lack of competence on a surgical task (53% of incidents), followed by communication breakdowns (43%) and fatigue or excessive workload (33%) [23]. As the newly incoming resident surgeon is expected to participate in relatively high-level and unfamiliar operations, more mistakes likely occur which are intercepted by the attending surgeon prior to any event of tangible operative consequence (i.e., intra- or post-operative complications). For single-console robotic surgery, time is significantly impacted when the attending must switch places with the resident seated on the console to resolve a mistake or misstep by the resident. Furthermore, various systematic factors in robotic surgery, such as a lapse in teamwork or communication breakdowns that affect the surgical flow, could have a greater-than-usual influence on surgery times during this particular resident transition period. For example, flow disruptions (deviations in the natural progression of a surgical task) are increased in robotic cases with residents [24, 25].

Our data revealed that residents introduced significant variability to ST when we compared the different operative time intervals across cases stratified by complexity (Fig. 1a). ST remained stable in cases with attending surgeons only (prior to the implementation of our residency program in 2011). These findings might be partially explained by differences in the surgical technical skills of the incoming residents. Factors such as manual precision and dexterity, as well as responsiveness to operative instruction, are not readily weighed in the residency match process compared to metrics of academic performance (i.e., percentile class ranking, involvement with research) [26], which potentially leads to considerable differences in the technical skillset among the incoming residents. Additionally, the variation in ST significantly related to resident involvement suggests that the degree of resident impact on operative time may vary by case complexity. Radical cystectomies (Group 3) resulted in the greatest ST difference when comparing times with and without trainees, and they are the most technically challenging group of cases in this study. Increased complexity inherently comes with more attending-to-resident intraoperative education that contributes to the total operative time. However, the impact of resident trainee presence on high-complexity cases is likely to be more nuanced. A 2017 review found that the learning curve for robotic-assisted cystectomies was shorter compared to the learning curve for robotic-assisted laparoscopic prostatectomies (a lower complexity case) [27], which is likely due to the fact that trainees most often take on complex cases only after attaining competency in cases that are determined to be less technically challenging. Therefore, technical inexperience in high-complexity cases may not be a substantial contributing factor to delayed operative time.

Another notable finding is the reduced non-OR time (NT) due to resident involvement by an estimated 4.6 min *(p* < *0.001)*. The small, yet significant, shortened time could possibly reflect improved workflow when it comes to accomplishing surgical tasks that are more standard and consistently performed across surgical cases. This can be understood by looking more closely at the processes that occur during NT. Time from operating room entry to initial incision was shorter, possibly reflecting a trainee’s contribution to the surgical team with proper patient positioning, foley catheter insertion, and preparing the patient in the usual sterile fashion. Time also improved from completion of the procedure to exit from the operating room, a finding which could reflect contributions to the turnover process, such as transferring the patient. Our results demonstrate that the inefficiencies found in the operative time due to resident involvement may be partially offset with an improved non-operative time, but unanswered questions remain in understanding the factors that affect operative times when residents are involved.

While any combination of the above could account for the complex, likely multifactorial, explanation behind the findings of the current study, our finding of significant operative delay associated with residents in the earlier months of the academic year should prompt consideration of interventions and changes for RAS training. Recently, the use of virtual reality (VR) simulation has been gaining traction to attempt shortening the robotic surgery learning curve without compromising patient safety [28]. Randomized trials have found improved RAS skills and performance in the operating room with VR-simulated practice [29]. Formally integrating VR simulation into training may allow for junior residents to practice surgical skills in a more controlled environment. Developing a means to objectively evaluate RAS trainees in a simulated setting is a key step to validating such curricula and incorporating them into surgical training programs.

### Limitations

The associations between operative variables and outcomes presented here do not necessarily prove causality. The variable-outcome association can be confounded by patient comorbidities and the operative experience of the resident surgeon. The multivariable models used in this study factored in patient age, BMI, case complexity, and attending surgeon experience; however, we were not able to adjust for resident experience, largely due to a limitation of the database concerning large inconsistencies with recording post-graduate year (PGY) level. Another limitation lies with using appropriate measure(s) for surgical outcomes, which often vary by the operation performed [30]. Besides EBL, post-operative length of stay and complications have been used in some studies and should be analyzed as an extension of this study. Lastly, our study was limited to a single center, but we suspect that there is broad generalizability to other institutions with similar practices.

## Conclusion

We found that resident involvement in U-RAS is associated with prolonged operative time, with the greatest delay occurring in cases performed from July to October. There may be a July Effect on operative and healthcare efficiency for patients undergoing urologic RAS early in the academic year. Cases with residents after October (cases from November to June) demonstrated significant improvement in the overall surgical duration, suggesting that the laparoscopically naïve resident surgeons become well-acclimated to robotic surgery and the overall surgical flow. Proactively addressing the training needs of the incoming residents might mitigate their impact on surgical times; further investigations are needed to evaluate any targeted interventions.

## Data Availability

Deidentified data that support the findings of this study are available from the corresponding author, upon reasonable request.

## References

[1] Durkin MJ, Dicks KV, Baker AW et al (2015) Seasonal variation of common surgical site infections: does season matter? Infect Control Hosp Epidemiol 36(9):1011–1016 26008876 10.1017/ice.2015.121 PMC4748703

[2] Honeyford K, Cecil E, Lo M, Bottle A, Aylin P (2018) The weekend effect: does hospital mortality differ by day of the week? A systematic review and meta-analysis. BMC Health Serv Res 18(1):870 30458758 10.1186/s12913-018-3688-3 PMC6245775

[3] Liu L, Hao D, Liu W, Wang L, Wang X (2020) Does weekend hospital admission affect upper gastrointestinal hemorrhage outcomes?: a systematic review and network meta-analysis. J Clin Gastroenterol 54(1):55–62 30119093 10.1097/MCG.0000000000001116

[4] Lagergren J, Mattsson F, Lagergren P (2017) Prognosis following cancer surgery during holiday periods. Int J Cancer 141(10):1971–1980 28730678 10.1002/ijc.30899

[5] Burke LG, Frakt AB, Khullar D, Orav EJ, Jha AK (2017) Association between teaching status and mortality in US hospitals. JAMA 317(20):2105–2113 28535236 10.1001/jama.2017.5702 PMC5815039

[6] Bahl A, Hixson CC (2019) July phenomenon impacts efficiency of emergency care. West J Emerg Med 20(1):157–162 30643619 10.5811/westjem.2018.10.39885 PMC6324718

[7] Young GJ, Charns MP, Daley J et al (1997) Best practices for managing surgical services: the role of coordination. Health Care Manag Rev 22(4):72–81 10.1097/00004010-199710000-00010 9358262

[8] Chan AK, Patel AB, Bisson EF et al (2021) “July Effect” revisited: july surgeries at residency training programs are associated with equivalent long-term clinical outcomes following lumbar spondylolisthesis surgery. Spine 46(12):836–843 33394990 10.1097/BRS.0000000000003903

[9] Marchegiani G, Andrianello S, Nessi C et al (2020) Seasonal variations in pancreatic surgery outcome a retrospective time-trend analysis of 2748 Whipple procedures. Updates Surg 72(3):693–700 32816284 10.1007/s13304-020-00868-6 PMC7481160

[10] Sharif-Afshar AR, Wood LN, Bresee C et al (2017) Teaching mid-urethral sling surgery to residents: impact on operative time and postoperative outcomes. Neurourol Urodyn 36(8):2148–2152 28370305 10.1002/nau.23259

[11] Coombs DM, Ascha MS, Ascha M et al (2020) Revisiting the “July Effect” in plastic surgery: new data to support resident autonomy. Ann Plast Surg 84(1):95–99 31688117 10.1097/SAP.0000000000001999

[12] Jena AB, Sun EC, Romley JA (2013) Mortality among high-risk patients with acute myocardial infarction admitted to U.S. teaching-intensive hospitals in july: a retrospective observational study. Circulation 128(25):2754–2763 24152859 10.1161/CIRCULATIONAHA.113.004074 PMC4125575

[13] Young JQ, Ranji SR, Wachter RM et al (2011) “July Effect”: impact of the academic year-end changeover on patient outcomes: a systematic review. Ann Intern Med 155(5):309–315 21747093 10.7326/0003-4819-155-5-201109060-00354

[14] Shah AA, Zogg CK, Nitzschke SL et al (2016) Evaluation of the perceived association between resident turnover and the outcomes of patients who undergo emergency general surgery: questioning the july phenomenon. JAMA Surg 151(3):217–224 26536282 10.1001/jamasurg.2015.3940

[15] ACGME (2020) A.C.f.G.M.E. Index Categories and Minimum Procedure Numbers http://acgme.org/Portals/0/PFAssets/ProgramResources/480-Urology-Case-Log-Info.pdf.

[16] Bravi CA, Larcher A, Capitanio U et al (2021) Perioperative outcomes of open, laparoscopic, and robotic partial nephrectomy: a prospective multicenter observational study (The RECORd 2 Project). EU Focus 7(2):390–396 31727523 10.1016/j.euf.2019.10.013

[17] Badani KK, Kaul S, Menon M (2007) Evolution of robotic radical prostatectomy: assessment after 2766 procedures. Cancer 110(9):1951–1958 17893904 10.1002/cncr.23027

[18] Bakaeen FG, Huh J, LeMaire SA et al (2009) The July Effect: impact of the beginning of the academic cycle on cardiac surgical outcomes in a cohort of 70,616 patients. Ann Thorac Surg 88(1):70–75 19559195 10.1016/j.athoracsur.2009.04.022

[19] Matulewicz RS, Pilecki M, Rambachan A et al (2014) Impact of resident involvement on urological surgery outcomes: an analysis of 40,000 patients from the ACS NSQIP database. J Urol 192(3):885–890 24704012 10.1016/j.juro.2014.03.096

[20] Ruhotina N, Dagenais J, Gandaglia G et al (2014) The impact of resident involvement in minimally-invasive urologic oncology procedures. Can Urol Assoc J 8(9–10):334–340 25408800 10.5489/cuaj.2170 PMC4216291

[21] Bedaiwy MA, Abdelrahman M, Deter S et al (2012) The impact of training residents on the outcome of robotic-assisted sacrocolpopexy. Minim Invasive Surg. 10.1155/2012/289342 23209891 10.1155/2012/289342 PMC3504428

[22] Casp AJ, Patterson BM, Yarboro SR et al (2018) The effect of time during the academic year or resident training level on complication rates after lower-extremity orthopaedic trauma procedures. J Bone Joint Surg Am 100(22):1919–1925 30480596 10.2106/JBJS.18.00279

[23] Gawande AA, Zinner MJ, Studdert DM et al (2003) Analysis of errors reported by surgeons at three teaching hospitals. Surgery 133(6):614–621 12796727 10.1067/msy.2003.169

[24] Jain M, Fry BT, Hess LW et al (2016) Barriers to efficiency in robotic surgery: the resident effect. J Surg Res 205(2):296–304 27664876 10.1016/j.jss.2016.06.092 PMC5074561

[25] Catchpole KR, Hallet E, Curtis S et al (2018) Diagnosing barriers to safety and efficiency in robotic surgery. Ergonomics 61(1):26–39 28271956 10.1080/00140139.2017.1298845 PMC6010349

[26] Calhoun KH, Hokanson JA, Bailey BJ (1997) Predictors of residency performance: a follow-up study. Otolaryngol Head Neck Surg 116(6):647–651 29389276 10.1016/S0194-5998(97)70242-0

[27] Mazzon G, Sridhar A, Busuttil G et al (2017) Learning curves for robotic surgery: a review of the recent literature. Curr Urol Rep 18(11):89 28942572 10.1007/s11934-017-0738-z

[28] Grover S, Tan GY, Srivastava A et al (2010) Residency training program paradigms for teaching robotic surgical skills to urology residents. Curr Urol Rep 11(2):87–92 20425095 10.1007/s11934-010-0093-9

[29] Bric JD, Lumbard DC, Frelich MJ et al (2016) Current state of virtual reality simulation in robotic surgery training: a review. Surg Endosc 30(6):2169–2178 26304107 10.1007/s00464-015-4517-y

[30] Shuhaiber JH (2002) Quality measurement of outcome in general surgery revisited: commentary and proposal. Arch Surg 137(1):52–54 11772215 10.1001/archsurg.137.1.52

